# Genome-Wide Identification of the NAC Gene Family and Expression Analysis Under Contrasting Salinity in *Zostera marina* L.

**DOI:** 10.3390/plants15213440

**Published:** 2026-11-05

**Authors:** Lujie Xie, Jie Liu, Hui Zhang, Qingqing Wang, Pei Yu, Yuchun Li, Xinfang Lv

**Affiliations:** 1 Marine College, Shandong University, Weihai 264209, China; 202417737@mail.sdu.edu.cn (L.X.); 202538014@mail.sdu.edu.cn (J.L.); 202437755@mail.sdu.edu.cn (H.Z.); 202437756@mail.sdu.edu.cn (Q.W.); li_yuchun@sdu.edu.cn (Y.L.); 2 Shandong University-Australian National University (SDU-ANU) Joint Science College, Shandong University, Weihai 264209, China; yupei@sdu.edu.cn

**Keywords:** eelgrass, NAC, gene family, abiotic stress, genome-wide identification

## Abstract

Eelgrass (*Zostera marina* L.) is a marine angiosperm exposed to substantial salinity fluctuations, yet the transcriptional mechanisms associated with its salinity adaptation responses remain incompletely understood. To characterize the NAC transcription factor family and its potential involvement in salinity responses, we performed genome-wide identification, phylogenetic, structural, duplication, promoter, and expression analyses in *Z. marina*, followed by quantitative reverse transcription PCR (qRT-PCR) validation. Sixty-three *ZmNAC* genes were identified and classified into six phylogenetic groups. Most encoded proteins contained a conserved N-terminal NAC domain but varied considerably in length, motif composition, gene structure, and predicted subcellular localization. Duplication analysis classified 55 genes as dispersed duplicates, seven as whole-genome duplication (WGD) or segmental duplicates, and one as a proximal duplicate. Promoter analysis identified 64 *cis*-acting element types associated mainly with light, phytohormone, and stress responses. RNA-seq analysis of roots, stems, and leaves after 24 h at NaCl concentrations of 0‰, 24‰, and 40‰ revealed distinct salinity- and tissue-dependent expression patterns among the *ZmNAC* family members. Expression patterns of four candidate genes in leaves, assessed by qRT-PCR, were largely consistent with the RNA-seq results. These findings reveal structural and expression diversification within the *ZmNAC* family and identify candidate genes for further functional investigation of salinity responses in eelgrass.

## 1. Introduction

Salinity stress profoundly affects plant growth and development, and suitable salinity conditions are essential for normal growth, development, and reproduction [1,2,3,4,5,6]. Salinity stress affects plant growth and development through the combined effects of osmotic stress, ion toxicity, oxidative damage, metabolic disruption, nutrient imbalance, and photosynthetic inhibition [2,3,4,5,6,7]. Severe stress can ultimately lead to plant death. Physiological adaptation, metabolic regulatory networks, and the mechanisms underlying salt tolerance have been extensively investigated in terrestrial plants [7,8,9,10]. However, the molecular basis of environmental adaptation in marine angiosperms remains less well characterized [11,12,13,14,15,16].

Eelgrass (*Zostera marina* L.), a representative member of the family Zosteraceae within the order Alismatales, is an important perennial seagrass widely distributed in the Northern Hemisphere [17,18,19]. In coastal and estuarine habitats, precipitation, river discharge, and surface runoff can cause rapid change in salinity [20]. Reduced salinity can affect eelgrass survival, growth, photosynthesis, and physiological performance, making hyposalinity an ecologically relevant challenge [21]. Examining responses to both increased and decreased salinity can therefore improve our understanding of eelgrass responses to salinity fluctuations. As one of the few angiosperms to have returned to the sea, *Z. marina* underwent an evolutionary transition from terrestrial to marine environments. This secondary colonization of marine habitats was accompanied by the loss of genes associated with terrestrial plants, including those involved in ethylene biosynthesis and stomatal development [12]. Genome remodeling also contributed to distinctive mechanisms of salt tolerance and adaptation to marine environments [13]. Recent whole-genome sequencing and comparative genomic studies have further revealed adaptive evolutionary features, including the sulfation of cell wall components and structural optimization of photosynthetic membrane proteins [12]. These studies have also highlighted functional divergence within key gene families, including Chalcone Synthase (CHS) and chloroplast NADH dehydrogenase-like (NDH) complex [14,15,16,17]. However, the transcriptional regulatory networks underlying eelgrass responses to changing salinity remain incompletely understood.

Transcription factors (TFs) are proteins that regulate target gene expression by binding to *cis*-acting elements in target gene promoters. TFs such as AP2, bHLH, ARF, MYC, WRKY, and NAC serve as essential regulators of numerous biological processes [22]. The NAC family is one of the largest plant-specific transcription factor families and has important roles in plant growth, development, and stress responses [23]. NAC proteins typically comprise a conserved N-terminal NAC domain and a highly variable C-terminal transcriptional regulatory region. The NAC domain contains approximately 150 amino acids and is divided into five subdomains (A–E). This domain primarily mediates DNA binding, protein dimerization, and nuclear localization [24,25]. By contrast, the C-terminal transcriptional regulatory region exhibits substantial sequence diversity and is a key determinant of transcriptional activation or repression [23,26]. NAC TFs regulate downstream gene expression by recognizing and binding to NAC recognition sequences (NACRSs) or NAC binding sites (NACBSs) in promoters of target genes. They participate in diverse biological processes, including organ development, lateral root formation, leaf senescence, xylem development, seed maturation, and hormone signaling [27,28]. They also have important regulatory roles in responses to abiotic and biotic stresses, including salinity, drought, cold, heat, flooding, and pathogen infection [29,30,31].

Numerous studies have shown that NAC transcription factors coordinate adaptive responses to salt stress by regulating ion homeostasis, osmolyte accumulation, reactive oxygen species (ROS) scavenging, and phytohormone signaling pathways, including abscisic acid (ABA) signaling [31,32,33,34]. Several NAC members regulate the expression of ion transport-related genes, including those in the Salt Overly Sensitive (SOS) pathway and the Na^+^/H^+^ antiporter (NHX) family, thereby contributing to cellular ion homeostasis [33]. NAC transcription factors also promote the accumulation of osmolytes, such as proline and soluble sugars, and enhance antioxidant enzyme activities. These enzymes include superoxide dismutase (SOD), peroxidase (POD), and catalase (CAT), which contribute to ROS scavenging and increased salt tolerance [31,33]. Transcriptomic and population-genomic studies in other seagrasses have identified tissue-specific regulatory programs and candidate loci associated with development or environmental adaptation [35,36], underscoring the need to examine specific transcription factor families in marine angiosperms.

Genome-wide identification and functional characterization of the NAC gene family have been conducted in diverse terrestrial plants, including *Arabidopsis* (*Arabidopsis thaliana*) [37], rice (*Oryza sativa*) [38], soybean (*Glycine max*) [39], maize (*Zea mays*) [40], and cotton (*Gossypium* spp.) [41]. The important roles of NAC genes in salt tolerance, drought tolerance, and adaptation to other environmental stresses have been widely demonstrated [33]. A broad comparative survey reported 62 NAC members in *Z. marina*, but did not examine the regulatory roles in marine environmental adaptation, particularly under high salinity, also lack systematic investigation [42]. To address these gaps, we used whole-genome data to identify NAC transcription factors in *Z. marina* and conduct comprehensive bioinformatic analyses. We aimed to characterize the evolutionary features of the *Z. marina* NAC gene family and explore its potential roles in adaptation to the marine environment. This study provides a foundation for functional validation of NAC genes and further investigation of the molecular regulatory mechanisms underlying salt tolerance in *Z. marina*.

## 2. Results

### 2.1. Identification of the ZmNAC Family Genes

**Figure 1:**
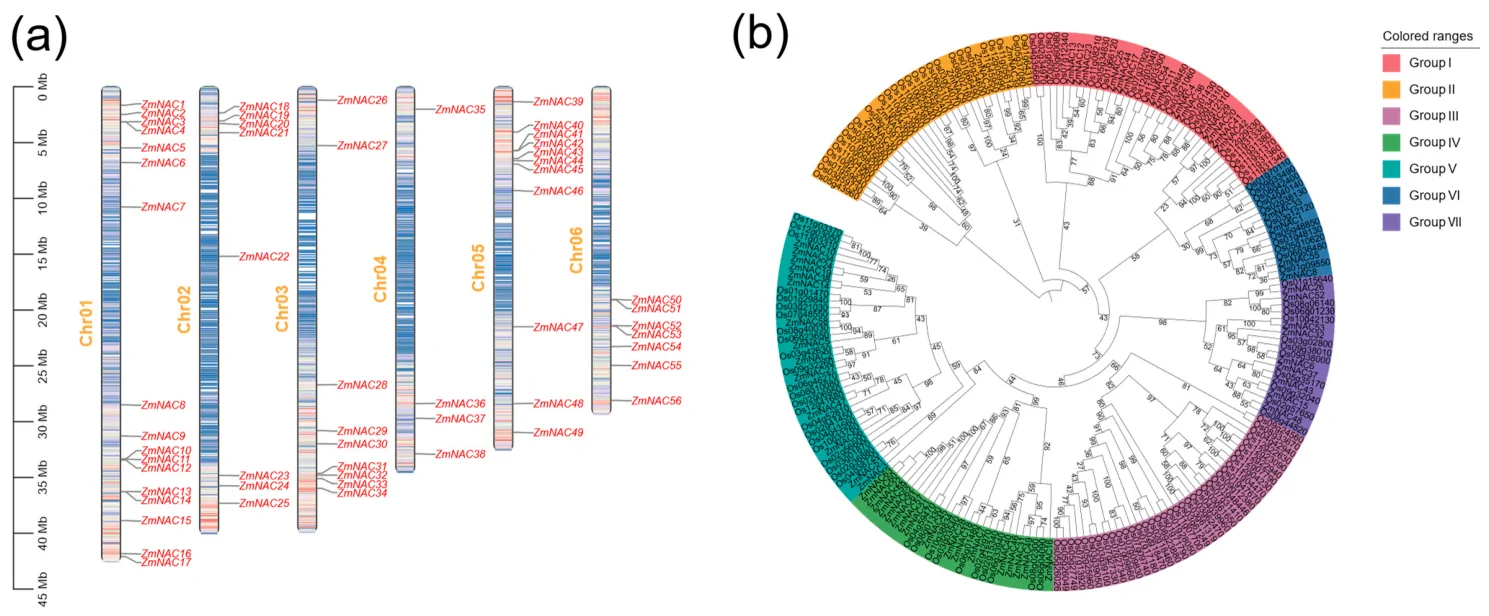
Chromosomal distribution of *ZmNAC* genes and combined phylogenetic analysis of NAC proteins from *O. sativa* subsp. *japonica* and *Z. marina*. (**a**) Chromosomal distribution of *ZmNAC* genes. The colored bands indicate gene density, with red and blue representing high and low gene densities, respectively. The scale on the left indicates chromosome length (Mb). *ZmNAC* genes are labeled according to their physical positions on the corresponding chromosomes. (**b**) Combined phylogenetic analysis of NAC proteins from *O. sativa* subsp. *japonica* and *Z. marina*. Roman numerals I–VII indicate the seven phylogenetic groups, and different colored regions represent distinct groups. Numbers at internal nodes indicate ultrafast bootstrap support values (%) based on 1000 replicates.

A total of 63 NAC genes were identified in *Z. marina* through BLAST and HMMER searches (PF02365) in TBtools v2.485, followed by validation against Pfam (Table S1). The genes were named *ZmNAC1*-*ZmNAC63* according to their positions, with unanchored genes listed after chromosome-assigned members. They were unevenly distributed across the genome, with the largest number on chromosome 1 (Figure 1a); *ZmNAC57*-*ZmNAC63* were located on unanchored scaffolds. Most candidates showed strong NAC/NAM-related HMM matches, although domain completeness varied.

A phylogenetic tree was constructed from the 63 ZmNAC protein sequences using IQ-TREE. Based on the evolutionary tree branches, ZmNAC proteins were classified into six phylogenetic groups (Groups I–VI; Figure S1). Group I, II, III, IV, V, and VI contained 14, 5, 9, 17, 14, and 4 genes, respectively. To further examine the evolutionary relationships between eelgrass and japonica rice NAC proteins, a combined phylogenetic tree was constructed using 63 ZmNAC and 141 rice NAC protein sequences. Based on the evolutionary tree branches, these proteins were classified into seven phylogenetic groups (Figure 1b). Groups I–VI corresponded to the six groups in the tree based solely on *Z. marina* and contained NAC members from both species, whereas Group VII contained only rice NAC members.

The 63 predicted ZmNAC proteins ranged from 66 to 1196 amino acids, with 24 members containing 300–400 amino acids (Table S2). Molecular weights ranged from 7667.76 to 134,379.92 Da, and theoretical isoelectric points varied from 4.14 to 9.90, indicating diverse acid-base properties. Instability indices ranged from 26.77 to 59.43. 26 ZmNAC proteins had values below 40 and were predicted to be relatively stable, whereas 37 ZmNAC proteins had values of 40 or higher. The aliphatic index ranged from 44.43 to 115.00, and GRAVY values ranged from −1.048 to 0.102. GRAVY was negative for 59 proteins, indicating that these proteins are predominantly hydrophilic. The four exceptions were ZmNAC22, ZmNAC59, ZmNAC60, and ZmNAC63. Subcellular localization analysis indicated that most ZmNAC proteins were predicted to be localized to the nucleus, whereas some members were predicted to be localized to the cytoplasm, chloroplast, peroxisome, and mitochondria (Table S2).

ORF integrity analysis showed that all 63 *ZmNAC* coding sequences contained canonical ATG start codons and in-frame termination codons, with no internal premature stop codons detected. Translation of the coding sequences using the standard genetic code yielded protein sequences identical to the corresponding annotated proteins. The five atypical gene models (*ZmNAC22*, *ZmNAC37*, *ZmNAC59*, *ZmNAC60*, and *ZmNAC63*) also exhibited intact annotated ORFs. However, these findings do not exclude potential gene annotation errors, and the structural reliability of these atypical models requires further evaluation. Therefore, these five genes were temporarily marked as low-confidence in Table S2. To assess whether they affected the family-level conclusion, we reconstructed the phylogenetic tree after excluding all five atypical models. The resulting topology and principal grouping pattern were unchanged, supporting the robustness of the main family-level conclusion (Figure S2).

### 2.2. Conserved Motifs, Domain and Gene Structure Analyses of the ZmNAC Family Genes

**Figure 2:**
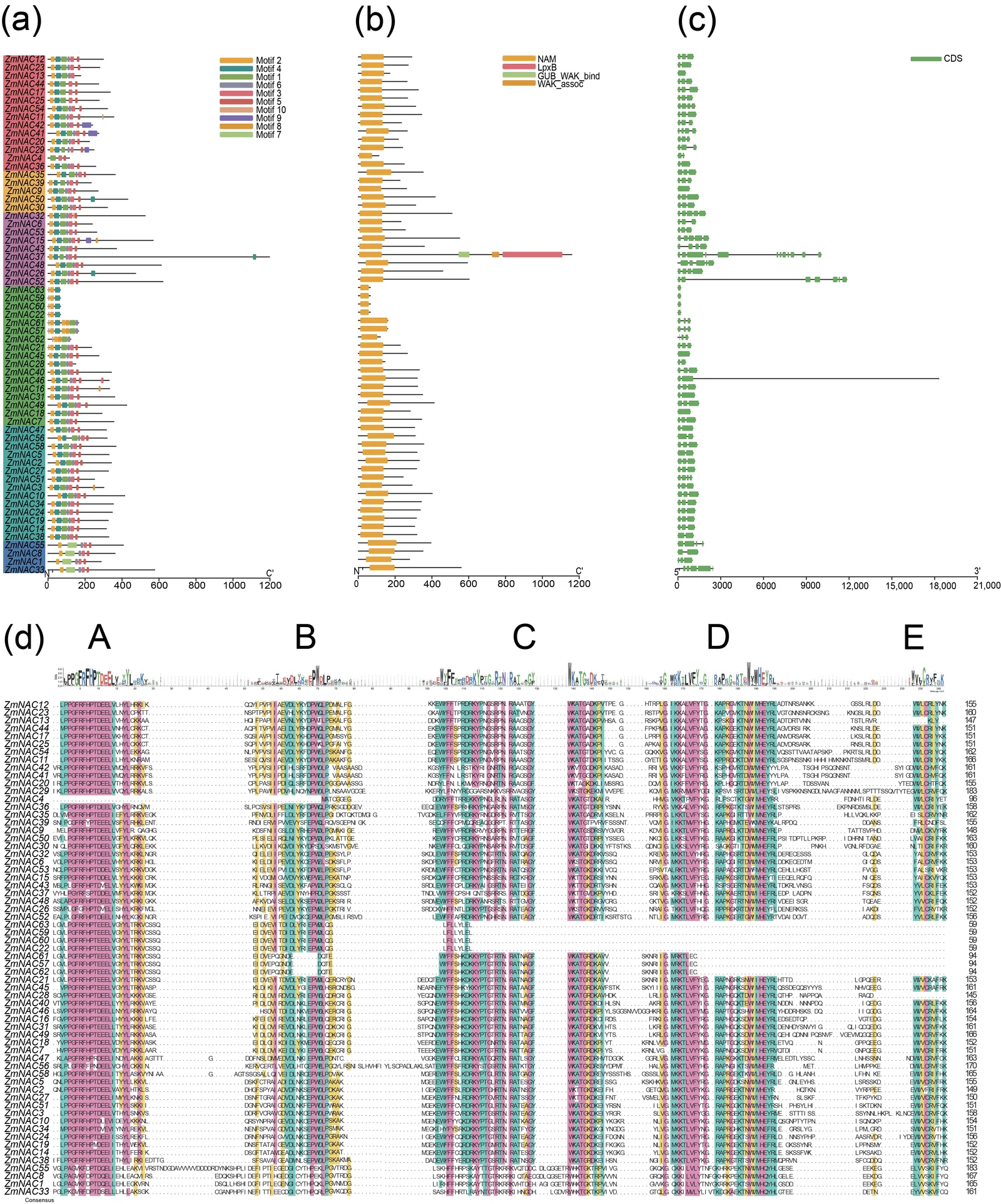
Protein and gene structures of *ZmNAC* family members. (**a**) Conserved motifs of ZmNAC proteins. (**b**) Domain organization of ZmNAC proteins. (**c**) Gene structures of *ZmNAC* genes. (**d**) Multiple sequence alignment of the NAC domain-containing regions of ZmNAC proteins. The alignment was displayed within an approximately 240 aa window spanning the NAC-domain-containing region, with shorter sequences represented by gaps as appropriate. Uncolored positions indicate relatively low amino acid similarity (<33%) among the ZmNAC proteins.

Conserved motifs analysis using MEME identified multiple motifs in ZmNAC proteins (Figure 2a). Motifs 1–6 were present in most members and were primarily concentrated in the N-terminal region, substantially overlapping the conserved NAC domain (Figure 2b). NAC/NAM-related signatures were detected in all retained candidates, although domain completeness and architecture varied. The C-terminal region exhibited considerable sequence variation, suggesting a potential contribution to functional divergence within the *ZmNAC* gene family. Exon structure analysis revealed considerable variation among *ZmNAC* genes, with the number of exons ranging from 1 to 12 (Figure 2c). Notably, members of Group III generally contained more exons than those in other groups. Multiple sequence alignment further delineated five NAC subdomains (A–E, Figure 2d), and most ZmNAC proteins retained relatively complete conserved N-terminal regions.

### 2.3. Gene Duplication Events and Collinearity Analyses of the ZmNAC Family Genes

**Figure 3:**
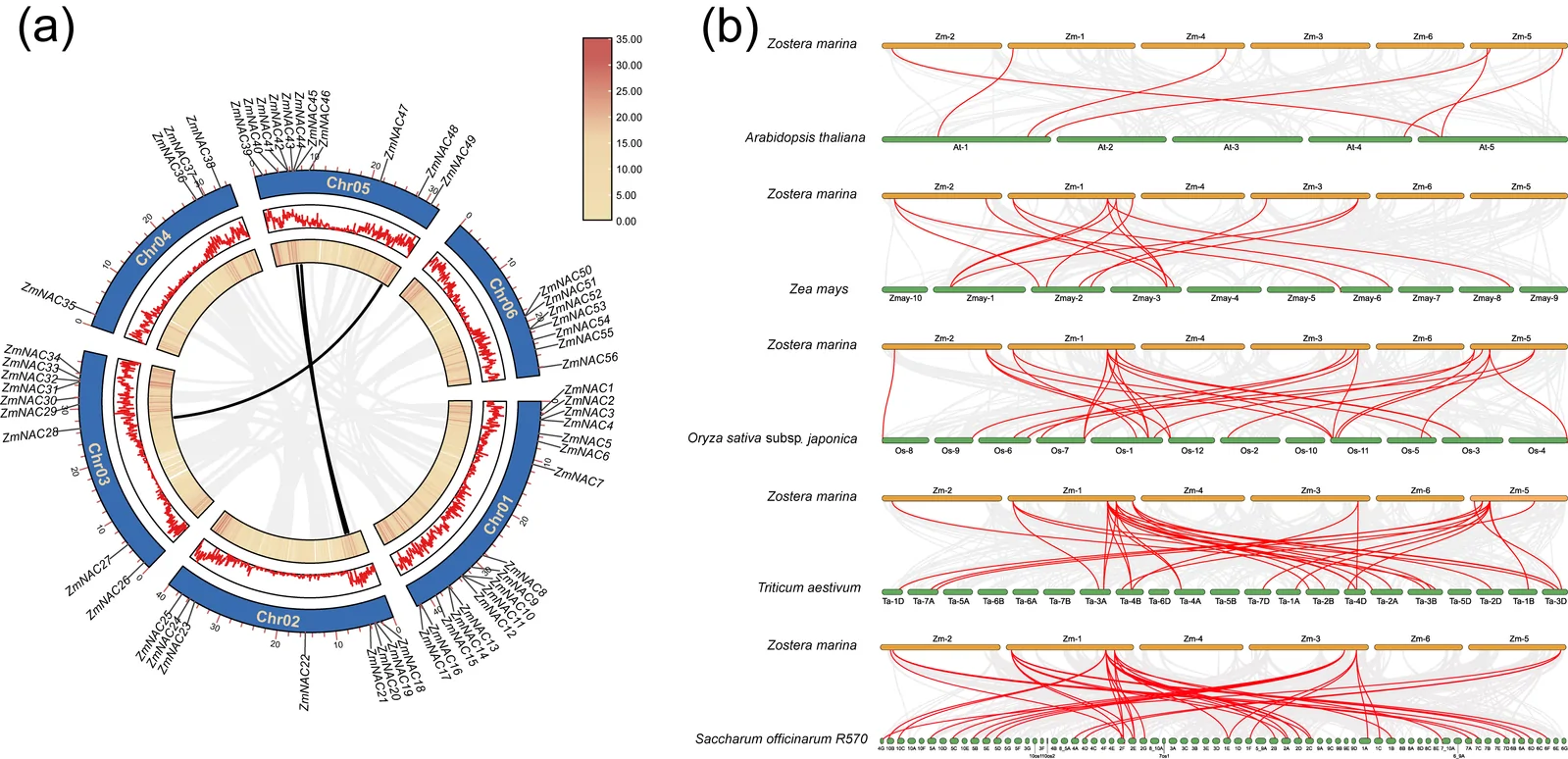
Collinearity analyses of the *ZmNAC* gene family. (**a**) Intraspecific collinearity analysis of the *ZmNAC* gene family. Black lines indicate collinear relationships involving *ZmNAC* genes. The blue rectangles in the outermost ring represent chromosomes, and the inner region shows gene density distribution. (**b**) Collinearity analyses of the NAC gene family between *Z. marina* and *A. thaliana*, *Z. mays*, *O. sativa* subsp. *japonica*, *T. aestivum*, and *S. officinarum* R570. Gray lines in the background represent collinear blocks between the *Z. marina* genome and the genomes of the other plant species, while red lines indicate collinear gene pairs.

Intraspecific collinearity analysis was performed to investigate the contribution of gene duplication to the evolution of the *ZmNAC* family. Three collinear gene pairs were identified: *ZmNAC21*-*ZmNAC45*, *ZmNAC20*-*ZmNAC41*, and *ZmNAC28*-*ZmNAC49* (Figure 3a). Extensive genome-wide collinear relationships were observed among chromosomes, including Chr2, Chr3, and Chr4.

To further investigate evolutionary conservation, interspecific collinearity analyses were conducted between *Z. marina* and *A. thaliana*, *Z. mays*, *O. sativa* subsp. *japonica*, *T. aestivum*, and *S. officinarum* R570 (Figure 3b). These comparisons identified 6, 15, 30, 35, and 38 collinear pairs, involving 6, 11, 16, 14, and 10 distinct *ZmNAC* genes, respectively (Tables S3–S7). Pair counts exceeded the numbers of distinct *ZmNAC* genes where a gene had multiple collinear counterparts. These *ZmNAC* genes were mainly located on chromosomes 1, 2, 3, and 5.

The 63 *ZmNAC* genes were classified into three duplication categories: dispersed, WGD or segmental, and proximal (Table S8). 55 genes were classified as dispersed duplicates and seven as WGD or segmental duplicates. Only *ZmNAC42* was classified as a proximal duplicate, and no tandem duplicates were identified. Thus, dispersed duplication was the most prevalent duplication category within the *ZmNAC* family. Ka/Ks analysis yielded ratios of 0.21069, 0.15197, and 0.089283 for the collinear gene pairs *ZmNAC20*-*ZmNAC41*, *ZmNAC21*-*ZmNAC45*, and *ZmNAC28*-*ZmNAC49* respectively (Table 1). All Ka/Ks ratios were below 1, consistent with predominantly purifying selection acting on these duplicated gene pairs.

### 2.4. Analysis of cis-Acting Elements in ZmNAC Family Gene Promoters

**Figure 4:**
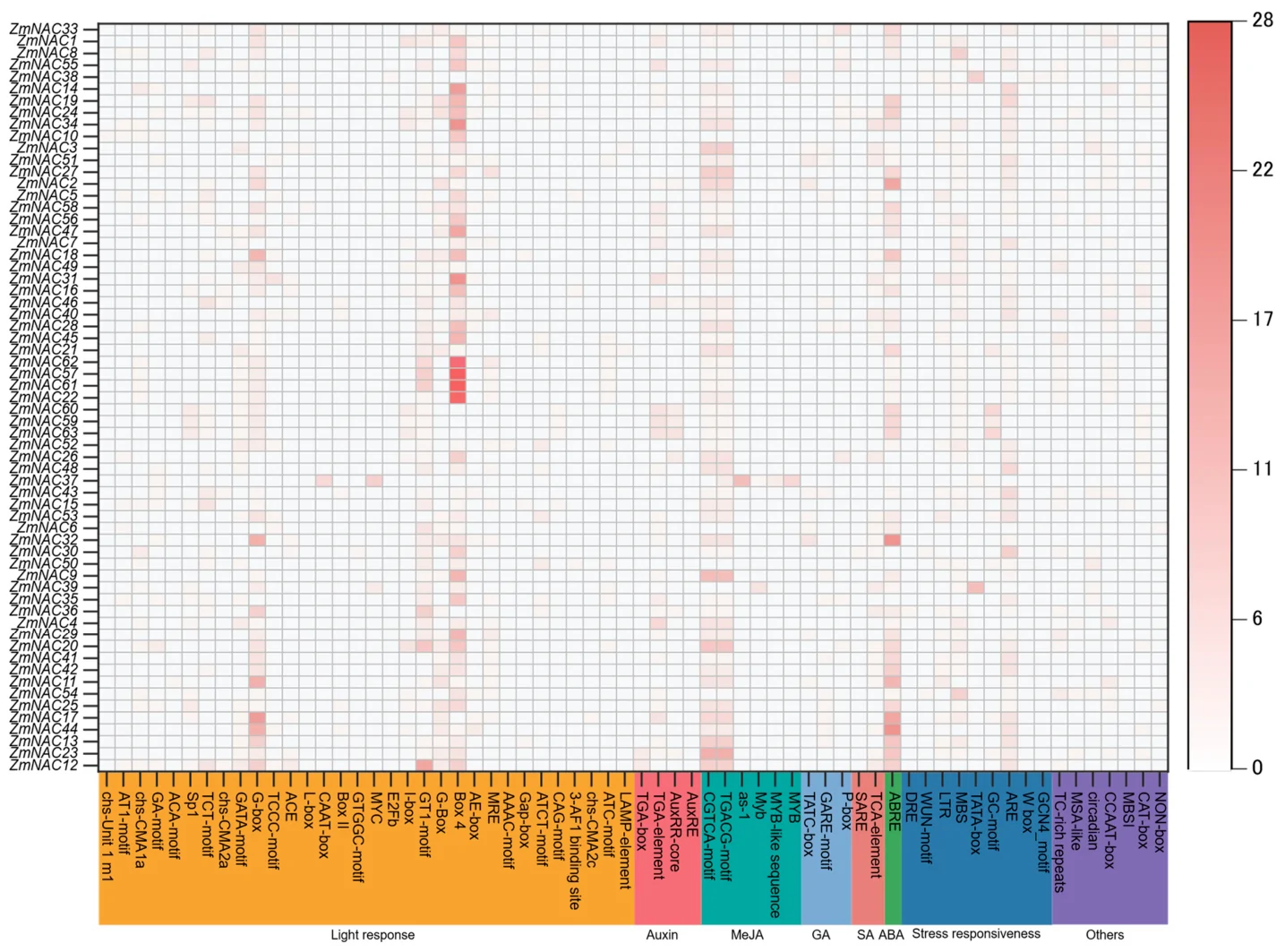
Heatmap of *cis*-acting elements in *ZmNAC* family genes. Color intensity represents the number of *cis*-acting elements, and different colors indicate different types of *cis*-acting elements.

To investigate the potential transcriptional regulation of *ZmNAC* genes, promoter sequences were extracted and analyzed for *cis*-acting regulatory elements. The occurrence and distribution of *cis*-regulatory elements were summarized descriptively. A total of 64 *cis*-acting element types associated with 31 putative regulatory functions were identified and classified into four major categories: light responsiveness, phytohormone responsiveness, stress responsiveness, and other growth- and development-related processes (Figure 4 and Figure S3).

Light-responsive elements were highly diverse, such as G-box, GT1-motif, and Box 4. Hormone-responsive elements were associated with auxin, methyl jasmonate, gibberellin, salicylic acid, and abscisic acid signaling. A total of 176 putative ABRE occurrences were detected among the *ZmNAC* promoters, representing one of the most frequently detected hormone-responsive element types in this dataset (Table S9). Stress-responsive elements included DRE, WUN-motif, LTR, MBS, ARE, and TC-rich repeats among others. In addition, several MYB-binding elements, including MBS, MRE, and MBSI, were detected. The composition and occurrence of predicted *cis*-acting elements varied among *ZmNAC* promoters, indicating heterogeneity in their putative regulatory architectures.

### 2.5. Expression Analysis of ZmNAC Family Genes Under Contrasting Salinity Conditions

**Figure 5:**
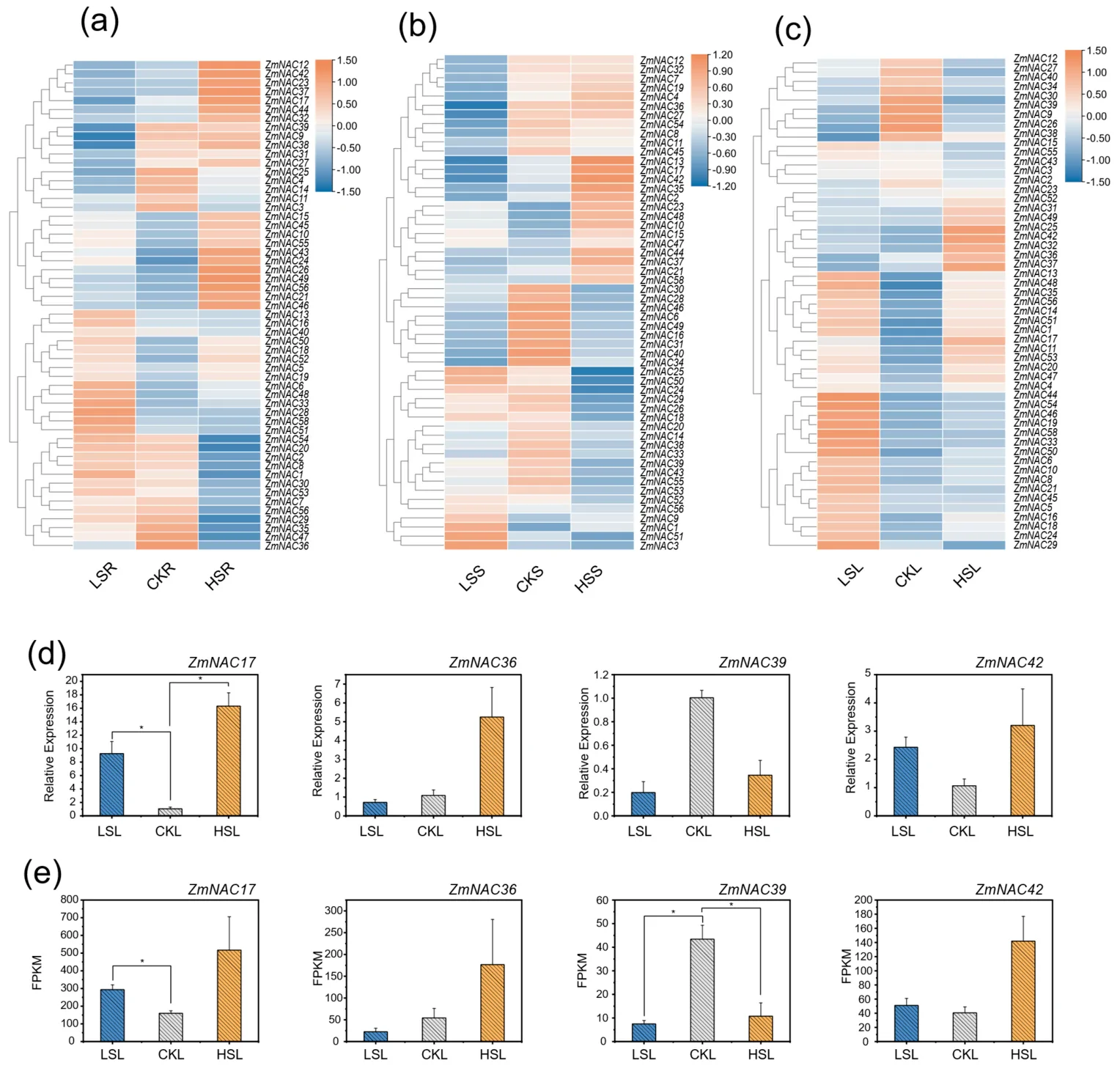
Expression patterns of *ZmNAC* family genes in different tissues under contrasting salinity treatments and qRT-PCR validation of four candidate genes. (**a**) Expression patterns of *ZmNAC* genes in roots under low-salinity (LSR), high-salinity (HSR), and control (CKR) conditions. (**b**) Expression patterns in stems under low-salinity (LSS), high-salinity (HSS), and control (CKS) conditions. (**c**) Expression patterns in leaves under low-salinity (LSL), high-salinity (HSL), and control (CKL) conditions. Yellow and blue indicate relatively high and low expression levels, respectively, and white indicates intermediate expression levels. (**d**) Relative expression levels determined by qRT-PCR. (**e**) FPKM values obtained from RNA-seq data. Data are presented as mean ± SE. Statistical significance was assessed using a two-tailed Student’s *t*-test (* *p* < 0.05).

RNA-seq analysis (Tables S10 and S11) revealed variation in *ZmNAC* expression among roots, stems, and leaves after salinity treatment (Figure 5a–c). Heatmaps show row-wise Z-scores derived from FPKM values. Replicate-level FPKM values are provided in Table S12, and sample relationships are shown by PCA and sample-to-sample distances (Figures S4 and S5). The genes displayed in the heatmap are those with nonzero expression levels and reliable cross-version correspondence.

Under high-salinity treatment, *ZmNAC17*, *ZmNAC23*, and *ZmNAC42* showed higher relative expression trends than the corresponding controls across all three tissues in the FPKM-based heatmaps (Figure 5a–c). Of these, *ZmNAC17*, *ZmNAC23*, and *ZmNAC42* belonged to Group I. Overall, Group I members showed predominantly higher relative expression in roots and stems, whereas Group II members showed mostly lower relative expression. *ZmNAC44* also showed higher relative expression than the corresponding control in roots and stems under high salinity, indicating a tissue-dependent response.

Under low-salinity treatment, *ZmNAC1*, *ZmNAC18*, *ZmNAC24*, and *ZmNAC50* showed higher relative expression than the corresponding controls across all three tissues in the FPKM-based heatmaps (Figure 5a–c). These patterns indicate distinct salinity- and tissue-associated expression profiles within the *ZmNAC* family after 24 h of salinity treatment.

Leaf qRT-PCR analysis of *ZmNAC17*, *ZmNAC36*, *ZmNAC39*, and *ZmNAC42* in an independent set of plants subjected to the same salinity treatments showed expression patterns largely consistent with RNA-seq (Figure 5d,e). Raw qRT-PCR output and the ΔCt, ΔΔCt, and relative-expression calculations are provided in Tables S13 and S14.

## 3. Discussion

NAC transcription factors participate extensively in plant growth, development, hormone signaling, and abiotic stress responses, forming an important component of regulatory networks underlying environmental adaptation [23]. The 63 *ZmNAC* genes identified here provide a basis for examining structural diversity and tissue-associated salinity responses in *Z. marina*. The slight difference from the 62 members reported previously [42] may reflect differences in genome annotation versions and gene-identification procedures. In addition, the *Z. marina* genome annotation has been updated from v2.1 to v3.1, which can alter gene models, merge or split gene loci, and change the total number of predicted protein-coding genes.

To account for differences in genome-wide gene content among species, NAC family size was additionally normalized to the number of protein-coding genes in the corresponding genome annotations. *Z. marina* contained approximately 2.93 NAC candidates per 1000 protein coding genes (63/21,483), compared with approximately 3.83 in *A. thaliana* (105/27,416) [37] and 3.75 in *Z. mays* (148/39,498) [40]. Thus, the family was smaller than those reported in terrestrial plants such as *A*. *thaliana*, *Z. mays*, both in absolute size and after normalization to protein-coding gene counts. These comparisons suggest differences in NAC representation among lineages. Beyond differences in genome size and ploidy, this variation may also be related to the distinctive evolutionary history of the *Z. marina*. *Z. marina* evolved from terrestrial angiosperm ancestors that subsequently recolonized marine environments, undergoing extensive genomic changes about marine adaptation. Previous genomic studies of *Z. marina* have revealed that several genes associated with terrestrial life have been lost or modified, such as stomatal development, volatile terpene biosynthesis, and UV protection [12,16]. Genes associated with cell walls, ion transport, and osmotic regulation were also modified during adaptation to seawater [12]. Therefore, the relatively small number of NAC family members in eelgrass may be related to its unique genomic evolutionary history.

ZmNAC proteins generally retained the characteristic structural features of NAC transcription factors (Figure 2). Most members contained a relatively conserved N-terminal NAC domain, where motifs 1–6 were concentrated, with their C-terminal regions showing considerable sequence variation. This pattern is consistent with the conserved N-terminal DNA-binding domain and variable C-terminal transcriptional regulatory region characteristic of NAC proteins [23,25]. Members in the same group generally shared similar motif compositions and gene structures (Figure 2), supporting consistency between phylogenetic relationships and structural features. Nevertheless, considerable variation in exon number and protein length was observed among *ZmNAC* genes and their encoded proteins (Figure 2c), suggesting that structural diversification has occurred despite conservation of the core NAC architecture. In *Arabidopsis*, Jensen et al. [26] showed that NAC proteins, including ANAC019, possess conserved DNA-binding domains could differ in binding affinity and transcriptional regulatory properties. Thus, conserved NAC architecture can coexist with functional specificity, although the functions of individual ZmNAC proteins remain to be established.

Some ZmNAC proteins exhibited differences in protein length or domain completeness compared with most family members. For example, ZmNAC22, ZmNAC59, ZmNAC60, and ZmNAC63 proteins were only approximately 66 aa (Table S2), and several other members exhibited varying degrees of subdomain loss. Comparable structural variation has been reported in NAC families from plants such as *Brachypodium distachyon* [43]. Following gene duplication, one copy may retain the ancestral function, while the other experiences relax selective pressure and are more likely to accumulate mutations such as deletions, insertions, and premature stop codons [44]. Over time, these changes may produce proteins lacking parts of their domains. Continued accumulation of disruptive mutations may compromise coding capacity, resulting in pseudogenes or truncated genes [45]. Innan and Kondrashov also proposed that one duplicate may retain the ancestral function while another copy acquires a new function through mutation, or the two copies may keep separately the partial functions of the ancestral gene [46]. Thus, partial structural loss does not necessarily result in complete protein inactivation. Instead, it may alter DNA binding, protein interactions, subcellular localization, or transcriptional activation or repression, potentially generating distinct regulatory properties [23,26,33]. Such changes may contribute to functional diversification within transcription factor families. But these may be truncated models or annotation artefacts.

The conserved N-terminal NAC domain typically contains nuclear localization signals (NLSs), which are recognized by the nuclear transport machinery to facilitate proteins import into the nucleus [23,25]. After entering the nucleus, NAC proteins recognize specific *cis*-acting elements in the promoters of target genes through their DNA-binding domains and activate or repress target gene expression via their C-terminal transcriptional regulatory regions [26]. The predicted nuclear localization of most ZmNAC proteins is consistent with their molecular roles as transcription factors (Table S2) [23]. However, NAC proteins are not exclusively localized to the nucleus [31]. Some members may occur in the cytoplasm, at the plasma membrane, or in organelles (Table S2). In particular, membrane-bound NAC transcription factors (NTLs) contain a C-terminal transmembrane domain and are initially anchored to membranes. Induced by environmental or developmental signals, they were released and translocated into the nucleus to regulate the expression of target genes [33]. A minority of ZmNAC proteins were predicted to localize to the cytoplasm, chloroplasts, mitochondria, or other cellular compartments (Table S2). These members may participate in processes such as signal transduction, photosynthesis, or redox homeostasis. But predictions for nuclear and nonnuclear members require experimental confirmation.

Phylogenetic analysis classified NAC members into seven groups in the combined tree (Figure 1). The presence of the group that only contained *OsNAC* genes (Group VII) in the combined tree suggests lineage-specific expansion, retention, or loss during the evolution of these NAC families. However, the current evidence is insufficient to determine whether this difference was directly driven by *Z. marina* adaptation to marine environments. A relatively small NAC gene family has also been reported in *Nymphaea colorata* [47]. Broader sampling of other seagrasses and closely related species within Alismatales, together with genome-size- and gene-content-aware comparisons, will be necessary to determine whether aquatic lineages show consistent differences in the relative representation of NAC genes.

Gene duplication is an important source of expansion and functional divergence in plant transcription factor families [48,49]. Duplication-type analysis classified most *ZmNAC* genes as dispersed duplicates and a smaller number as WGD or segmental duplicates (Figure 3a). Thus, dispersed duplication played a predominant role in the evolution of the *ZmNAC* family. Dispersed duplication has also been reported as the principal driver of NAC family expansion in *Rhododendron delavayi* [50]. A comparative study of NAC families across 24 terrestrial plant species found that species with more WGD events generally possessed larger NAC families [51]. Differences in duplication and retention history may therefore help explain variation in NAC family size, but this requires further verification.

Collinear relationships were detected between *ZmNAC* genes and their counterparts in *A. thaliana*, *Z. mays*, *O. sativa* subsp. *japonica*, *T. aestivum*, and *S. officinarum* R570 (Figure 3b). More collinear gene pairs were generally identified with the sampled monocots than with *A. thaliana*, consistent with the phylogenetic placement of *Z. marina* within the monocots. Pair counts are also influenced by ploidy, duplication history, gene retention, and assembly and annotation quality. In particular, wheat is allohexaploid, sugarcane R570 has a complex polyploid hybrid genome [52,53,54]. *ZmNAC1* had collinear counterparts in all five species (Tables S3–S7). *ZmNAC10*, *ZmNAC12*, *ZmNAC13*, *ZmNAC14*, and *ZmNAC34* also had collinear homologs in multiple monocot species (Tables S4–S7). Undefined Ks or Ka/Ks estimates were reported as NaN and excluded from selection-pressure inference. The result of NaN may be due to the large evolutionary distance between sequences, which may cause saturation of synonymous sites, resulting in a synonymous replacement rate pS exceeding 0.75, reaching the computational boundary of the evolutionary model, leading to meaningless negative values after formula calculation.

Predicted promoter *cis*-acting element composition provides clues to the potential upstream regulation of *ZmNAC* genes. Numerous *cis*-acting elements associated with light, phytohormone, and environmental stress responses were identified in *ZmNAC* promoters (Figure 4). Light-responsive elements participate in regulating plant physiological processes such as light acclimation and photoperiod-dependent responses. In *N. colorata*, and several other species, light-responsive elements are the predominant *cis*-acting elements in NAC gene promoters [47,55,56,57]. Previous studies have found light-responsive elements are related to the increase in organic acid production and enhanced stress tolerance in *Nicotiana tabacum* L. [58]. In *A. thaliana*, *ANAC078* overexpression increased flavonoid biosynthesis under high light [59]. Light signaling has also been shown to regulate flavonoid metabolism through the photoreceptor-mediated COP1/SPA–HY5 pathway in *A. thaliana* [60]. NAC transcription factors in *A. thaliana*, *T. aestivum*, and other plants have been implicated in ABA-associated stress regulatory pathways [29,31,33]. These findings motivate investigation of light-related regulation in eelgrass, but whether the light-responsive elements upstream of the *ZmNAC* gene regulate flavonoid biosynthesis still needs further experimental verification. ABRE and other predicted hormone- and stress-responsive motifs also provide candidates for promoter assays. These predictions should be tested by promoter-reporter assays and functional studies.

RNA-seq analysis revealed distinct salinity- and tissue-dependent responses within the *ZmNAC* family (Figure 5a–c). Similar patterns have been reported in other plants. In *O. sativa*, different *OsNAC* genes had distinct responses to salt stress considerably. Under 100 mM NaCl treatment, some *OsNAC* genes were upregulated at 3 h but repressed at 6 h, whereas others showed the opposite patterns [61,62]. That suggests there was dynamic functional specialization among NAC family genes. In *Allium sativum*, *AsNAC* genes also exhibited distinct expression patterns at 1, 4, and 12 h after salt treatment. For example, *AsNAC29* responded early, whereas *AsNAC18* and *AsNAC20* reached higher expression levels at later stages; the expression patterns also differed between roots and leaves [63]. The induction of different gene sets under high and low salinity suggests that *Z. marina* may employ distinct NAC regulatory modules to respond to increasing and decreasing salinity, rather than depending on a single graded response (Figure 5a–c). Expression patterns identified different candidates under high and low salinity. *ZmNAC17*, *ZmNAC23*, and *ZmNAC42* showed higher relative expression across all three tissues under high salinity, while *ZmNAC1*, *ZmNAC18*, *ZmNAC24*, and *ZmNAC50* showed this pattern under low salinity (Figure 5a–c). *ZmNAC1* is homologous to *A. thaliana SND3* (*AT1G28470.1*) (Table S3) which primarily participates in secondary cell wall formation in fiber and xylem cells, and its overexpression promotes secondary wall thickening in fiber cells [64]. Under low salinity, *ZmNAC1* showed higher relative expression in roots, stems, and leaves. Low salinity alters the osmotic balance across cell membranes and may lead to water influx and cell turgor. The induction of *ZmNAC1* may be associated with cell wall remodeling or the maintenance of cellular mechanical stability, a possibility that warrants further testing.

Duplicate genes did not always retain similar expression patterns. *ZmNAC28* and *ZmNAC49*, a collinear pair classified as WGD or segmental duplication type, showed contrasting salinity-dependent expression trends in roots (Figure 5). *ZmNAC28* showed its highest relative expression under low salinity, whereas *ZmNAC49* showed its highest relative expression under high salinity (Figure 5). Their promoters also differed in *cis*-acting element composition. The contrasting expression and promoter profiles suggest that the two genes may have developed distinct salinity responses following duplication while retaining some sequence and structural conservation [44,65]. Changes in upstream regulatory regions may have contributed to this regulatory divergence [66]. Interestingly, the expression of these two genes is consistent in the stem.

Phylogenetically related *ZmNAC* members did not necessarily exhibit identical responses to salinity stress. For example, *ZmNAC1*, *ZmNAC8*, *ZmNAC33*, and *ZmNAC55* (Group VI members) showed distinct tissue-associated expression patterns under high salinity (Figure 5a–c), and their predicted promoters differed in *cis*-acting element composition (Figure 4 and Figure S3). Similarly, the duplicated pair *ZmNAC28*-*ZmNAC49* exhibited contrasting salinity responses (Figure 5a–c). Such variation suggests that phylogenetic or structural conservation does not necessarily predict similar transcriptional responses and divergence in promoter regions following gene duplication may be associated with distinct salinity- and tissue-dependent responses among *ZmNAC* family genes [46,65]. *ZmNAC44* also showed higher relative expression in both roots and stems under high salinity. Its *Arabidopsis* homolog, *AT1G77450*.1, participates in salicylic acid/jasmonic acid signaling and root growth regulation [66,67], suggesting hormone-related regulation as a hypothesis for future investigation. The expression and comparative genomic evidence support prioritizing *ZmNAC44* and other responsive members for functional assays, while leaving their specific regulatory roles unresolved.

These findings suggest that *ZmNAC* family genes may contribute to salinity responses in eelgrass. However, the present study is based on a single 24 h treatment and sampling from one site and season, so the temporal dynamics of these responses and their generality under moderate natural salinity fluctuations remain to be determined. In addition, qRT-PCR validation was limited to leaves and a single reference gene, and the specific functions of these genes in different tissues have not yet been resolved. Future work should therefore incorporate multiple time points, a broader range of salinity conditions, and functional assays to systematically investigate the roles of *ZmNAC* genes in eelgrass salinity adaptation.

## 4. Materials and Methods

### 4.1. Identification of ZmNAC Family Genes

TBtools v2.485 was used to analyze protein physicochemical properties, perform hidden Markov model (HMM) searches, construct phylogenetic trees, analyze gene structures, and visualize results [68]. The *Z. marina* genome (v3.1) and its corresponding annotation file in GFF3 format were downloaded from Phytozomev14 (https://phytozome-next.jgi.doe.gov/ (accessed on 2 January 2026)) [69]. The *A. thaliana* genome (TAIR10) and its corresponding GFF3 annotation file were downloaded from The *Arabidopsis* Information Resource (TAIR) (https://www.arabidopsis.org/ (accessed on 2 January 2026)) [70]. The japonica rice (*O. sativa* subsp. *japonica*) genome (ASM3414082v1) and its corresponding annotation file were obtained from the National Center for Biotechnology Information (NCBI) (https://www.ncbi.nlm.nih.gov/ (accessed on 2 January 2026)). Genome sequences and corresponding annotation files for wheat (*Triticum aestivum* cv. Chinese Spring, v2.1), maize (*Z. mays*, RefGen_V4), and sugarcane (*Saccharum officinarum* × *S. spontaneum*, R570 v2.1) were downloaded from Phytozome.

NAC protein sequences from *A. thaliana* were used as queries to search the *Z. marina* protein database. Homology searches were performed using the BLAST function in TBtools v2.485, which invokes BLASTp, with an E-value threshold of <1 × 10^−5^ to identify initial candidates. In parallel, the HMM profile for the conserved NAC domain (PF02365) was downloaded from Pfam v38.2 (https://www.ebi.ac.uk/interpro/ (accessed on 2 January 2026)). This profile was used to search the *Z. marina* protein database using the Simple HMM Search module in TBtools [71]. Examination of the original HMM search output confirmed that this module executed HMMER hmmsearch v3.2.1 with its default search settings and without a user-defined HMM E-value threshold. The candidate sets obtained from the BLASTp and HMM searches were intersected, and duplicate gene entries within the overlapping candidate set were removed. The resulting nonredundant candidates were subsequently screened using InterPro v108.0 (https://www.ebi.ac.uk/interpro/ (accessed on 2 January 2026)) to verify NAC/NAM-related domain signatures, thereby defining the retained NAC-related candidate set in *Z. marina* [72]. The *ZmNAC* CDS nucleotide sequences are provided in Data S1, and the corresponding ZmNAC protein sequences are provided in Data S2.

The integrity of the open reading frames (ORFs) of the 63 ZmNAC genes was assessed using TBtools-II. The coding sequences (CDSs) were translated into protein sequences using the Batch Translate CDS to Protein function with the standard genetic code. The presence of canonical start codons (ATG), termination codons (TAA, TAG, or TGA), and internal premature stop codons was examined. The translated protein sequences were then compared with the annotated protein sequences to verify sequence consistency, with particular attention to five atypical gene models (ZmNAC22, ZmNAC37, ZmNAC59, ZmNAC60, and ZmNAC63).

### 4.2. Physicochemical Characterization of ZmNAC Proteins

The Protein Parameter Calc module in TBtools was used to characterize the physicochemical properties of NAC proteins in *Z. marina*. These properties included amino acid count, molecular weight, theoretical isoelectric point (pI), instability index, aliphatic index, and grand average of hydropathicity (GRAVY). Subcellular localization was predicted using the WoLF PSORTv0.2 (https://wolfpsort.hgc.jp/ (accessed on 4 January 2026)) web server, and all results were compiled [73].

### 4.3. Chromosomal Localization and Phylogenetic Analysis

Genome-wide gene density was calculated using the Gene Density Profile module in TBtools. NAC genes were mapped to their physical positions on the corresponding chromosomes or chromosome segments using the Gene Location Visualize from GTF/GFF module. A chromosomal distribution map was generated alongside the genome-wide gene density profile to examine the distribution patterns of NAC genes across chromosomes.

NAC protein sequences from *O. sativa* subsp. *japonica* were downloaded from PlantTFDB v4.0 (http://planttfdb.gao-lab.org/ (accessed on 1 January 2026)) [74]. The 141 rice NAC protein sequences were combined with the NAC protein sequences of the *Z. marina*. Multiple sequence alignment was performed using the MUSCLE Wrapper module in TBtools, and poorly aligned regions were removed using the trimAl Wrapper module with the ML_AUTOMATED1 trimming mode. A maximum-likelihood (ML) phylogenetic tree was then constructed using the IQ-TREE Wrapper module, with branch support assessed using 1000 ultrafast bootstrap replicates. The best fit amino acid substitution model was selected separately for each dataset by IQ-TREE according to the Bayesian Information Criterion (BIC). The phylogentic tree of ZmNAC proteins was inferred using JTTDCMut+G4, whereas the combined phylogenetic tree of NAC proteins from eelgrass and rice was inferred using JTT+R9. No external outgroup was specified during tree reconstruction; the resulting trees were subsequently midpoint-rooted in iTOL v7 (https://itol.embl.de/ (accessed on 5 May 2026)) for visualization [75]. The alignment and IQ-TREE output for the phylogeny of ZmNAC proteins are provided in Data S3 and Data S4, respectively; the alignment and IQ-TREE output for the phylogeny of NAC proteins from eelgrass and rice are provided in Data S5 and Data S6, respectively.

### 4.4. Conserved Motif, Domain, and Gene Structure Analyses

Conserved motifs in NAC protein sequences of the *Z. marina* were identified using the MEME Suite web server (Multiple Expectation Maximization for Motif Elicitation, https://meme-suite.org/meme/ (accessed on 6 March 2026)) in Classic mode with protein sequences as input. The site-distribution model was set to zero or one occurrence per sequence (ZOOPS), and the maximum number of motifs was set to 10. All other parameters were left at their default settings. The results were imported into TBtools to visualize motif composition and distribution alongside the phylogenetic relationships among NAC proteins [76].

Conserved protein domains were identified using the NCBI CD-Search service (https://www.ncbi.nlm.nih.gov/Structure/bwrpsb/bwrpsb.cgi (accessed on 8 March 2026)) [77]. The results were imported into TBtools to visualize conserved domain composition and distribution alongside the phylogenetic relationships among NAC proteins.

Gene structures were visualized using the Visualize Gene Structure module in TBtools, based on the *Z. marina* genome annotation file (GFF3) and NAC gene sequence information. Coding sequence (CDS) annotations were used to generate gene structure diagrams and compare coding region organization among family members. Protein multiple sequence alignment was performed using DNAMAN v8.0.8.789 (https://www.lynnon.com/ (accessed on 7 March 2026)). All 63 ZmNAC proteins alignment display was restricted to an approximately 240-aa window spanning the NAC-domain-containing region, with shorter sequences represented by gaps as appropriate. Sequence logos were generated using WebLogo 3 (https://weblogo.threeplusone.com/ (accessed on 8 March 2026)) [78].

### 4.5. Collinearity, Gene Duplication, and Selection Pressure Analyses

Collinearity analyses of the *ZmNAC* gene family were performed using TBtools. Comparative species included *A. thaliana*, *Z. mays*, *O. sativa* subsp. *japonica*, *T. aestivum*, and *S. officinarum* R570. Intraspecific collinearity of *Z. marina* was analyzed using the One Step MCScanX module, and the resulting collinear gene pairs, gene positions, chromosome lengths, and gene density profiles were integrated and visualized using the Advanced Circos module, with *ZmNAC* genes highlighted [79].

Interspecific collinearity between *Z. marina* and five comparative species and gene duplication events were analyzed using the One Step MCScanX-Super Fast module and visualized with the Dual Synteny Plot for MCScanX, with *ZmNAC* genes labeled to illustrate conserved syntenic relationships. Collinear gene pairs identified by MCScanX were subjected to pairwise Ka/Ks analysis using the Simple Ka/Ks Calculator module in TBtools. For each collinear gene pair, the corresponding coding sequences were used to estimate the nonsynonymous substitution rate (Ka), synonymous substitution rate (Ks), and Ka/Ks ratio.

### 4.6. Analysis of cis-Acting Elements in ZmNAC Gene Promoters

The 2000 bp sequence upstream of the translation initiation site (ATG) of each *ZmNAC* gene was defined as its promoter region. Promoter sequences were extracted from the *Z. marina* genome assembly (FASTA) using the corresponding GFF3 annotation file and the GXF Seq Extract module in TBtools using CDS as the feature tag and Parent as the feature identifier. Extraction followed gene orientation, including reverse-strand genes. A 2 kb upstream interval was requested for each target, and no additional neighboring-gene boundary filter or overlap-based truncation was applied. The extracted sequences were submitted to PlantCARE (https://bioinformatics.psb.ugent.be/webtools/plantcare/html/ (accessed on 11 January 2026)) for *cis*-acting element prediction [80]. The predicted elements were tabulated and summarized in Microsoft Excel, and the results were visualized using Origin V2024 10.1.0.178 SR1. The complete raw PlantCARE output is provided in Data S7. Only curated records assigned to the 64 cis-element types summarized in Table S9 were counted.

### 4.7. Plant Treatment and RNA-Seq Analysis of ZmNAC Genes

*Z. marina* plants were collected in May 2023 from Shuangdao Bay, Weihai, China (37°28′ N, 121°58′ E). Healthy plants with intact roots, stems, and leaves were transported to the laboratory, rinsed with artificial seawater, and acclimated for 72 h at 24‰ NaCl (30‰ total salinity, containing 24‰ NaCl and other dissolved salts). Artificial seawater was prepared following the general procedure described by Lv et al. [11], and the acclimation medium was replaced daily. Plants were then assigned to control (CK, 24‰ NaCl), low-salinity (LS, 0‰ NaCl), and high-salinity (HS, 40‰ NaCl) treatments under natural light at 20 °C with continuous aeration. For each salinity treatment, three independent treatment vessels were established, each containing three individual *Zostera marina* plants. After 24 h of treatment, root, stem, and leaf tissues were collected separately. For each tissue type, samples from the three plants within the same vessel were pooled to constitute one biological replicate, resulting in three independent biological replicates per treatment. The pooled tissue samples were immediately frozen in liquid nitrogen and stored at −80 °C until RNA extraction.

Total RNA was extracted using TRIzol reagent (Invitrogen, Shanghai, China). RNA quality was assessed using a NanoDrop 2000 spectrophotometer (Thermo Scientific, Wilmington, DE, USA) and an Agilent 2100 Bioanalyzer (Agilent Technologies, Santa Clara, CA, USA). RNA-seq libraries were prepared using the VAHTS Universal V5 RNA-seq Library Prep Kit (VAHTS, Nanjing, China) and sequenced on an Illumina NovaSeq 6000 platform(Illumina, San Diego, CA, USA) to generate 150 bp paired-end reads. Raw reads were filtered using fastp and aligned to the *Z. marina* reference genome (https://www.ncbi.nlm.nih.gov/search/all/?term=GCA_001185155.1_Zosma_marina.v.2.1 (accessed on 20 May 2023)) using HISAT2. Sequencing was performed by Qingdao OE Biotech Co., Ltd. (Qingdao, China), and the data were deposited in the NCBI Sequence Read Archive (SRA) under accession numbers SRR31298605–SRR31298629 and SRR31298638–SRR31298639. *ZmNAC* expression levels were calculated as fragments per kilobase of transcript per million mapped reads (FPKM), normalized to Z-scores, and visualized as heatmaps using TBtools. Replicate-level FPKM values are provided in Table S12. In the heatmap, we excluded genes with an expression level of 0 and those that do not have a reliable cross-version correspondence.

Gene-level raw read counts were obtained using HTSeq-count. Principal component analysis (PCA) and sample-to-sample distance analysis were conducted in R (v3.2.0) to assess sample relationships and reproducibility (Figures S4 and S5). The RNA-seq count matrix for all 27 samples is also provided in Table S15.

### 4.8. qRT-PCR Validation of ZmNAC Genes

Gene-specific primers were designed using Primer3 (https://primer3.ut.ee/ (accessed on 6 June 2026)) and synthesized by Sangon Biotech (Shanghai) Co., Ltd. (Shanghai, China) [81]. Primer sequences, design parameters, and expected amplicon lengths are provided in Table S16. First-strand cDNA was synthesized using TransScript^®^ All-in-One First-Strand cDNA Synthesis SuperMix for qPCR (One-Step gDNA Removal) (TransGen, Beijing, China) at 42 °C for 30 min followed by 85 °C for 1 min. qPCR was performed using PerfectStart^®^ Green qPCR SuperMix on an ABI QuantStudio^®^ Real-Time PCR System(Applied Biosystems, Waltham, MA, USA) with an initial denaturation at 94 °C for 30 s, followed by 45 cycles of 94 °C for 5 s and 60 °C for 30 s. Actin11 (ZOSMA_146G00440) was used as the reference gene. Three biological samples and two technical replicates per biological sample were analyzed for each treatment. The Ct values of the two technical replicates were averaged before ΔCt and ΔΔCt calculations. Relative expression levels were calculated using the 2^−ΔΔCt^ method and visualized using Origin [82], whereas statistical significance was assessed using a one-tailed Student’s *t*-test on ΔCt values from the three biological samples. The instrument output is provided in Table S13. The calculation process used to derive ΔCt, ΔΔCt, and relative-expression values is provided in Table S14.

## 5. Conclusions

This study characterized 63 NAC genes in *Z. marina*. The family showed conserved NAC structural features, a predominance of dispersed duplicates, and varied promoter and expression profiles. Under short-term salinity treatment, *ZmNAC17*, *ZmNAC23*, and *ZmNAC42* showed higher relative expression across roots, stems, and leaves under high salinity, whereas *ZmNAC1*, *ZmNAC18*, *ZmNAC24*, and *ZmNAC50* showed a similar trend under low salinity. These genes are candidates for functional investigation. Experiments with additional time points and representative salinity regimes are needed to test their roles in eelgrass salinity responses.

## Figures and Tables

**Table 1:** Ka/Ks ratios of intrafamily collinear *ZmNAC* gene pairs.

Seq_1	Seq_2	Ka	Ks	Ka/Ks
*ZmNAC20*	*ZmNAC41*	0.325416	1.544526	0.210690
*ZmNAC21*	*ZmNAC45*	0.248383	1.63442	0.151970
*ZmNAC28*	*ZmNAC49*	0.192564	2.156776	0.089283

Ka, nonsynonymous substitution rate; Ks, synonymous substitution rate. All Ka/Ks ratios were <1, indicating that the three duplicated *ZmNAC* gene pairs have predominantly undergone purifying selection. In the genome-wide MCScanX analysis, ZmNAC47 was paired with Zosma03g23910, but the latter was not classified as a member of the ZmNAC family because its domain annotations did not show a significant NAC/NAM domain. We therefore clarified that seven ZmNAC genes were assigned to the WGD/segmental category in the genome-wide classification, whereas only three pairs contain two retained ZmNAC members and are consequently listed as intrafamily pairs in Table 1.

## Data Availability

The RNA-sequencing data generated in this study are publicly available in the NCBI Sequence Read Archive (SRA; https://www.ncbi.nlm.nih.gov/sra (accessed on 2 January 2026)) under accession numbers SRR31298605-SRR31298629 and SRR31298638-SRR31298639. All other data supporting the findings of this study are available within the article and its Supplementary Materials. Further information is available from the corresponding author upon reasonable request.

## Supplementary Materials

The following supporting information can be downloaded at: https://www.mdpi.com/article/10.3390/plants15213440/s1, Figure S1: Phylogenetic tree of ZmNAC proteins; Figure S2: Phylogenetic tree of ZmNAC proteins excluding all five atypical models; Figure S3: *Cis*-acting elements of the *ZmNAC* gene family; Figure S4: Principal component analysis (PCA) of RNA-seq samples; Figure S5: Sample-to-sample distance heatmap; Table S1: Domain scores; Table S2: Prediction of physicochemical properties and subcellular localization of ZmNAC proteins; Table S3: Collinearity gene pairs and Ka/Ks ratios between the *A. thaliana* NAC gene family and the *ZmNAC* gene family; Table S4: Collinearity gene pairs and Ka/Ks ratios between the *Z. mays* NAC gene family and the *ZmNAC* gene family; Table S5: Collinearity gene pairs and Ka/Ks ratios between the *O. sativa* subsp. *japonica* NAC gene family and the *ZmNAC* gene family; Table S6: Collinearity gene pairs and Ka/Ks ratios between the *T. aestivum* NAC gene family and the *ZmNAC* gene family; Table S7: Collinearity gene pairs and Ka/Ks ratios between the *S. officinarum* R570 NAC gene family and the *ZmNAC* gene family; Table S8: Duplication types of *ZmNAC* gene family; Table S9: Gene-by-cis-acting-element count matrix of predicted cis-acting elements in the 2 kb upstream regions of *ZmNAC* genes; Table S10: RNA-seq sequencing and mapping statistics; Table S11: Sample-to-accession metadata; Table S12: Replicate-level FPKM expression matrix; Table S13: qRT-PCR instrument output and melting-temperature information; Table S14: qRT-PCR calculation process; Table S15: RNA-seq raw count matrix; Table S16: qRT-PCR primers and expected amplicon lengths; Data S1: *ZmNAC* CDS nucleotide sequences; Data S2: ZmNAC protein sequences in FASTA format; Data S3: ZmNAC multiple-sequence alignment; Data S4: IQ-TREE output for the phylogeny of ZmNAC proteins; Data S5: Combined multiple-sequence alignment of NAC proteins from *O. sativa* subsp. *japonica* and *Z. marina*; Data S6: IQ-TREE output for the combined phylogeny of NAC proteins from *Z. marina* and *O. sativa* subsp. *japonica*; Data S7: Raw Plant-CARE output.

## Abbreviations

The following abbreviations are used in this manuscript:

NAC	NAM, ATAF1/2, CUC2
WGD	Whole-genome duplication
ABREs	Abscisic acid-responsive elements
TFs	Transcription factors
AP2	APETALA2
bHLH	Basic helix–loop–helix
ARF	Auxin response factor
MYB	Myeloblastosis
ABA	Abscisic acid
pI	Theoretical isoelectric point
GRAVY	Grand average of hydropathicity
Ka	Nonsynonymous substitution rate
Ks	Synonymous substitution rate
