# Supplementary material for: Genome-Wide Identification of the NAC Gene Family and Expression Analysis Under Contrasting Salinity in Zostera marina L
Source: Plants (Basel). 2026 Nov 5;15(21):3440. doi: 10.3390/plants15213440 (PMC13766098; doi:10.3390/plants15213440)
Supplement: Supplementary file 1 [file plants-15-03440-s001.zip › Supplementary Figures.pdf]

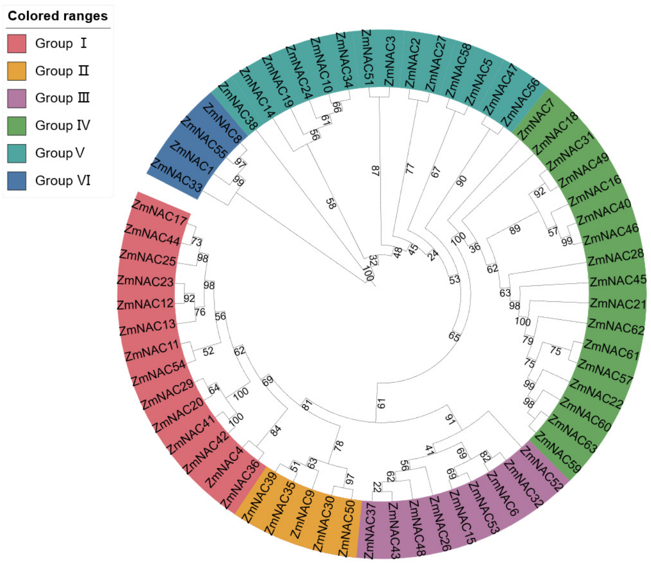

Figure S1. Phylogenetic tree of ZmNAC proteins. Numbers at internal nodes indicate ultrafast bootstrap support values (%) based on 1,000 replicates.

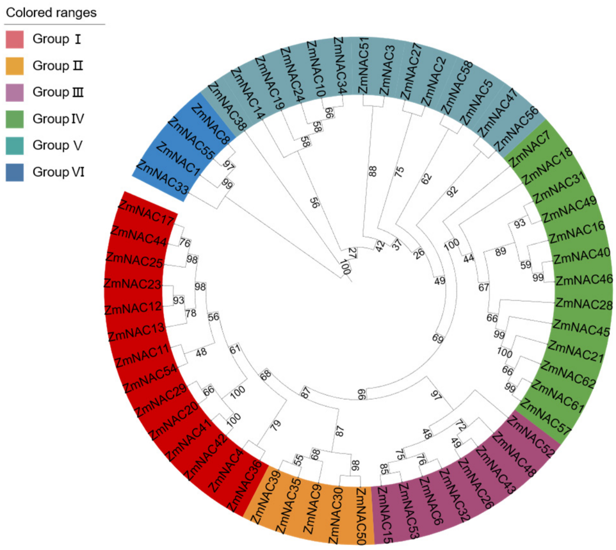

Figure S2. Phylogenetic tree of ZmNAC proteins excluding all five atypical models. Numbers at internal nodes indicate ultrafast bootstrap support values (%) based on 1,000 replicates.

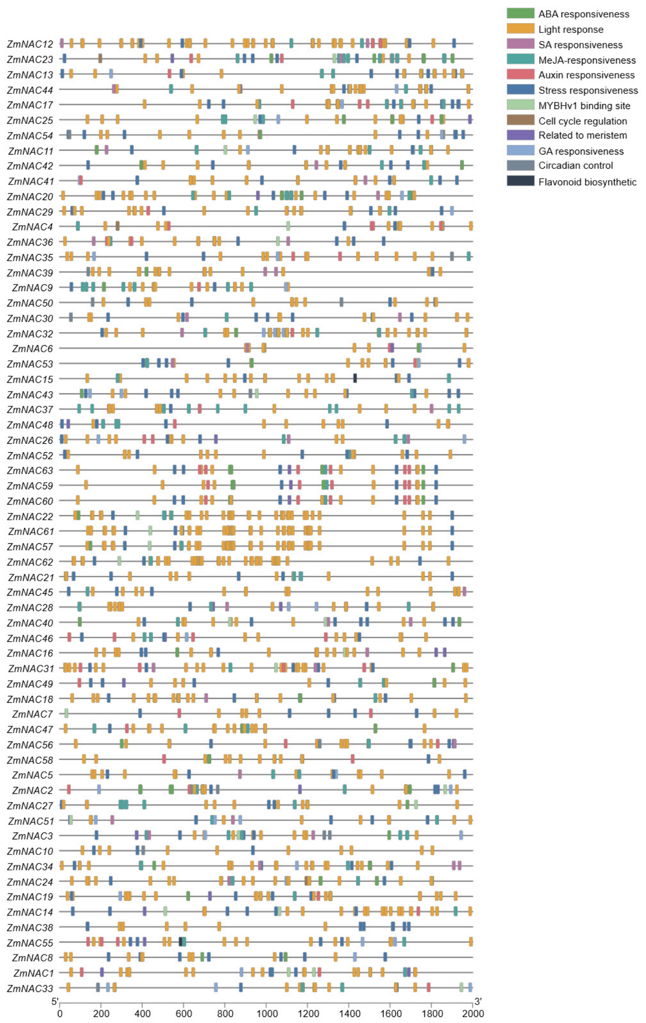

Figure S3. *Cis*-acting elements of the *ZmNAC* gene family.

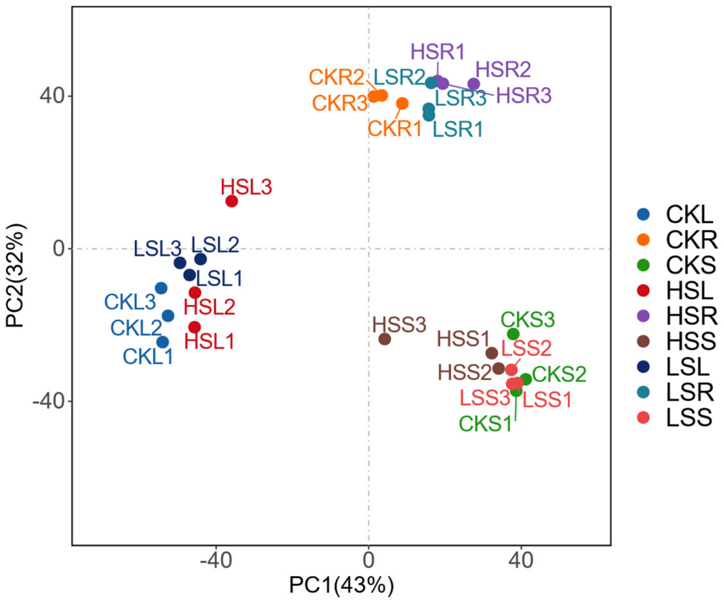

Figure S4. Principal component analysis (PCA) of RNA-seq samples.

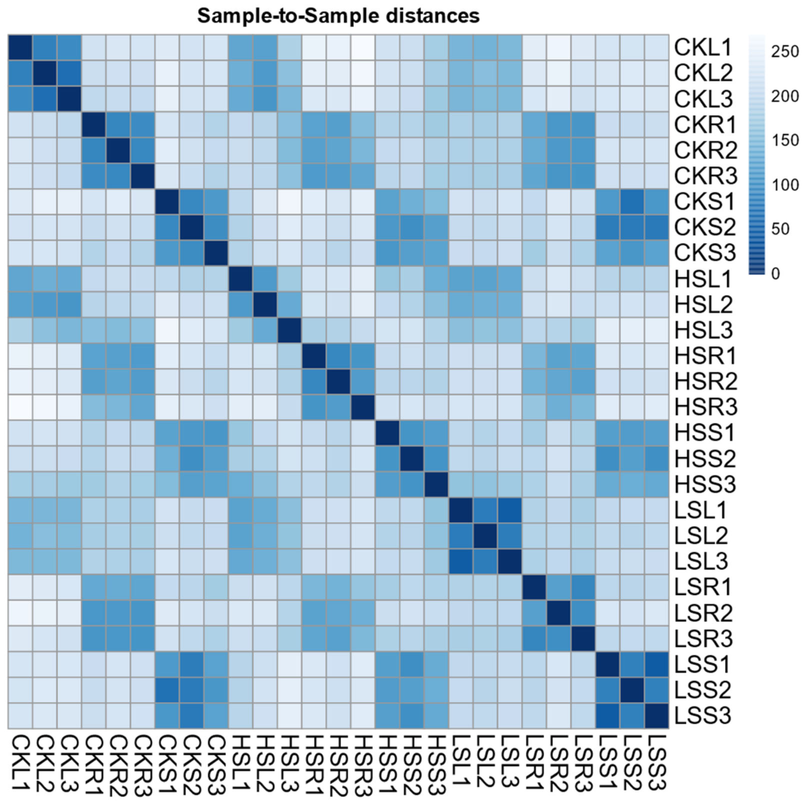

Figure S5. Sample-to-sample distance heatmap of RNA-seq samples.
